# Bisphosphonate treatment and dental implants: A systematic review

**DOI:** 10.4317/medoral.20920

**Published:** 2016-07-31

**Authors:** Nayara-Ribeiro de-Freitas, Lívia-Bonjardim Lima, Marcos-Boaventura de-Moura, Cizelene-do-Carmo-Faleiros Veloso-Guedes, Paulo-César Simamoto-Júnior, Denildo de-Magalhães

**Affiliations:** 1 Special student of the master’s program of the dental school of the Federal University of Uberlandia; 2 Regular student of the master’s program of the dental school of the Federal University of Uberlandia; 3 Regular student of the doctoral degree program of the dental school of the Federal University of Uberlandia; 4 Professor of the master’s program of the dental school of the Federal University of Uberlandia

## Abstract

**Background:**

To analyze articles that studied patients submitted to diphosphonates therapy and who received dental implants before, during or after bisphosphonate (BP) treatment, compared to healthy patients, analyzing the increase of failure and loss of implants or bisphosphonate related osteonecrosis of the jaw (BRONJ) incidence.

**Material and Methods:**

The Preferred Reporting Items for Systematic Reviews and Meta-analysis (PRISMA) statement was used in this study. The clinical question in “PICO” format was: In patients under bisphosphonate therapy, do dental implants placement, compared to healthy patients, increase the failure and loss of implants or bisphosphonate related osteonecrosis of the jaw incidence? PubMed/MEDLINE was searched for articles published up until April 15, 2015 using a combination of MeSH terms and their Entry terms.

**Results:**

The search resulted in 375 articles. After selection according to the eligibility criteria, 15 studies fulfilled were included (eight retrospective, one prospective and six case series), with a total of 1339 patients analyzed, 3748 implants placed, 152 loss of implants and 78 cases of BRONJ.

**Conclusions:**

Due to the lack of randomized clinical trials looking at this theme, further studies with longer follow-up are needed to elucidate the remaining questions. Thus, it is wise to be careful when planning dental implant surgery in patients undergoing bisphosphonate therapy because of the risk of developing BRONJ as well as occurring failure of implant. Moreover, complete systemic condition of the patient must be also taking into considering when such procedures are performed.

**Key words:**Bisphosphonates, diphosphonates, dental implants, osteonecrosis.

## Introduction

Bisphosphonates (BPs) are pyrophosphate analogues with high affinity for the bone hydroxyapatite. Due to their pharmacological effects on the bone, they play an important role on skeletal disorders with enhanced or imbalanced bone remodeling rates (1). They are considered effective drugs in treatment of disease affecting bone metabolism, characterized by increased resorption, such as osteoporosis, Paget’s disease, hypercalcemia of malignancy, multiple myeloma and bone metastasis of prostate, lung and breast cancer (2,3).

These drugs are divided into first-generation non-nitrogen-containing (clodronate, etidronate and tiludronate) and second and third generation nitrogen-containing (alendronate, risedronate, ibandronate and zoledronate) and the last ones differ from the others because they adhere more tightly to hydroxyapatite mineral in bone (1).

The route of administration affects the skeletal uptake of the medication. Oral bisphosphonates are poorly absorbed and present less than one percent of bioavailability, whereas the intravenous are completely bioavailable (1). Oral Bisphosphonates include alendronate, risedronate, etidronate, tiludronate. Pamidronate and zoledronate are only intravenous, whereas ibandronate and clodronate are administrated by both routes (4).

One of the most serious complications of BP therapy is Bisphosphonate Related Osteonecrosis of the Jaws (BRONJ). Because of the growing number of osteonecrosis cases in the jaws associated with other antiresorptive and antiangiogenic therapies, American Association of Oral and Maxillofacial Surgeons (AAOMS) in 2014 suggested a nomenclature change from BRONJ to Medication Related Osteonecrosis of the Jaw (MRONJ) (5).

Osteonecrosis induced by bisphosphonates is characterized by exposed bone or bone that can be probed through an intraoral or extraoral fistula in the maxillofacial region that has persisted for more than eight weeks in patients who have received current or previous treatment with antiresorptive or antiangiogenic agents and no history of radiation therapy to the jaws or metastatic disease to the jaws (5). Mandible and maxilla are bones exposed to the external environment, through the teeth. First cases of BRONJ were most likely associated to previously tooth removal surgery or other condition that increases the demand for bone turnover (6).

That is why there is controversy whether it is safe to place implants in patients taking bisphosphonates for bone diseases.

This review aimed to analyze articles that studied patients who were submitted to bisphosphonate therapy and who received dental implants before, during or after the BP treatment. The comparison was made with heathy patients who did not were under BP treatment and the outcomes observed were possible failures and loss of implants and the incidence of Bisphosphonate Related Osteonecrosis of the Jaws.

## Material and Methods

- Search Strategies

The PubMed-Medline database of the United States National Library of Medicine, National Institutes of Health, Bethesda, Maryland, was electronically searched for articles published up until April 15, 2015. The Preferred Reporting Items for Systematic Reviews and Meta-analysis (PRISMA) statement was used in this study (7). The clinical question in “PICO” format (*P* = patient problem / population, I = Intervention, C = Comparison, O = Outcome) in our study was:

In patients under bisphosphonate therapy, do dental implants placement, compared to healthy patients, increase the failure and loss of implants or bisphosphonate related osteonecrosis of the jaw incidence? 

The following MeSH (Medical Subjects Headings) terms: “Diphosphonates”, “Dental Implants”, “Guided Tissue Regeneration”, “Guided Tissue Regeneration, Periodontal”, “Alveolar Bone Grafting”, “Subgingival Curettage”, “Gingivectomy”, “Bisphosphonate-Associated Osteonecrosis of the Jaw” and their related entry terms were used in different combinations using the Boolean Operators “AND” and “OR” for the research. In addition, manual search was made by each one of the researchers.

Before starting the study, exclusion and inclusion criteria were established:

- Exclusion criteria:

(a) Articles published in another language other than English or Portuguese; (b) experimental laboratory studies; (c) animal studies; (d) studies that the main topic was not the relation between dental implants and systemic bisphosphonate therapy. (e) systematic reviews; (f) topical administration route of bisphosphonates; (g) full text articles were not available on the data base searched; (h) single case reports; (i) duplicated articles; (j) letters to editor; (k) commentaries.

- Inclusion criteria:

(a) Articles enrolled patients undergoing bisphosphonate therapy (oral and intravenous) and submitted to dental implants procedure; (b) case series; (c) retrospective Studies; (d) prospective Studies.

## Results

**Figure 1:**
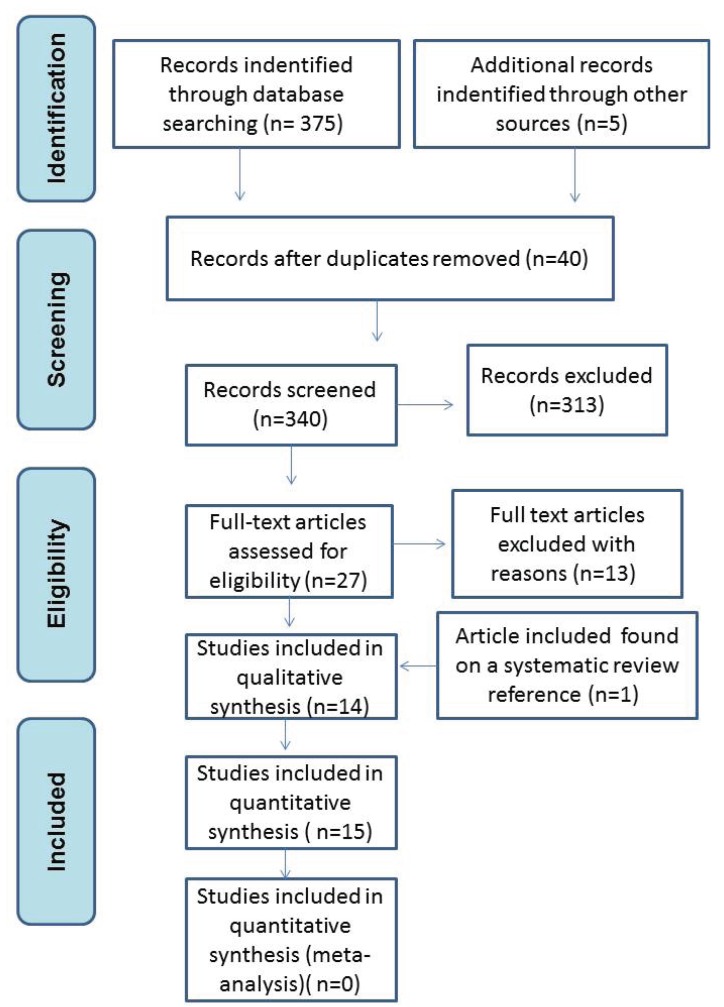
Prisma® flow diagram of the search processes and results.

The initial search resulted in a list of 375 articles. In turn, titles were analyzed and based on exclusion criteria only 152 abstracts were included. After reading of the available abstracts, 27 articles were read and two of them were excluded because it was observed that they were not in accordance with the inclusion criteria described next, and eight systematic reviews were used only as a research source. Finally, 17 articles were assessed for data extraction. After the final evaluation 3 papers were excluded because they did not focus on the relationship between dental implants and bisphosphonate therapy or their sample enrolled much more patient that were not under bisphosphonate treatment than patient under bisphosphonate therapy. Additionally, one article was included after manual search. Lastly, data from 15 studies fulfilled the inclusion criteria and were used to compose this systematic review (Fig. 1).

The data presented in  Table 1, Table 2: Author and year; gender of patients; age of patients (years); risk factors; number of patients in the study; number of implants placed; number of loss of implants; follow-up period (months); indication, type of bisphosphonate used and route of administration; duration of BP treatment (months); number and location of BRONJ were extracted from the 15 selected studies.

**Table 1:**
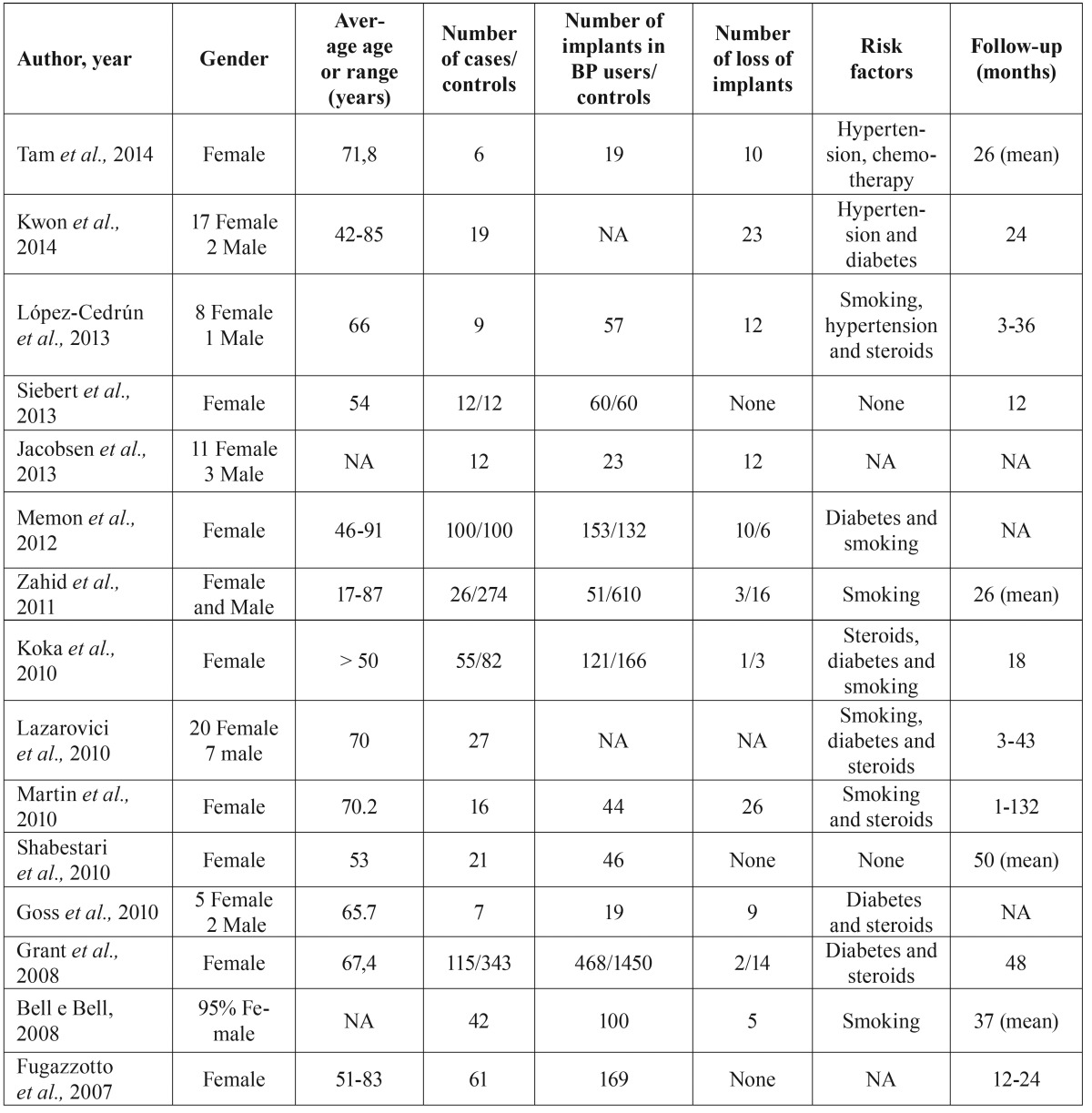
Summary of the studies meeting the eligibility criteria.

**Table 2:**
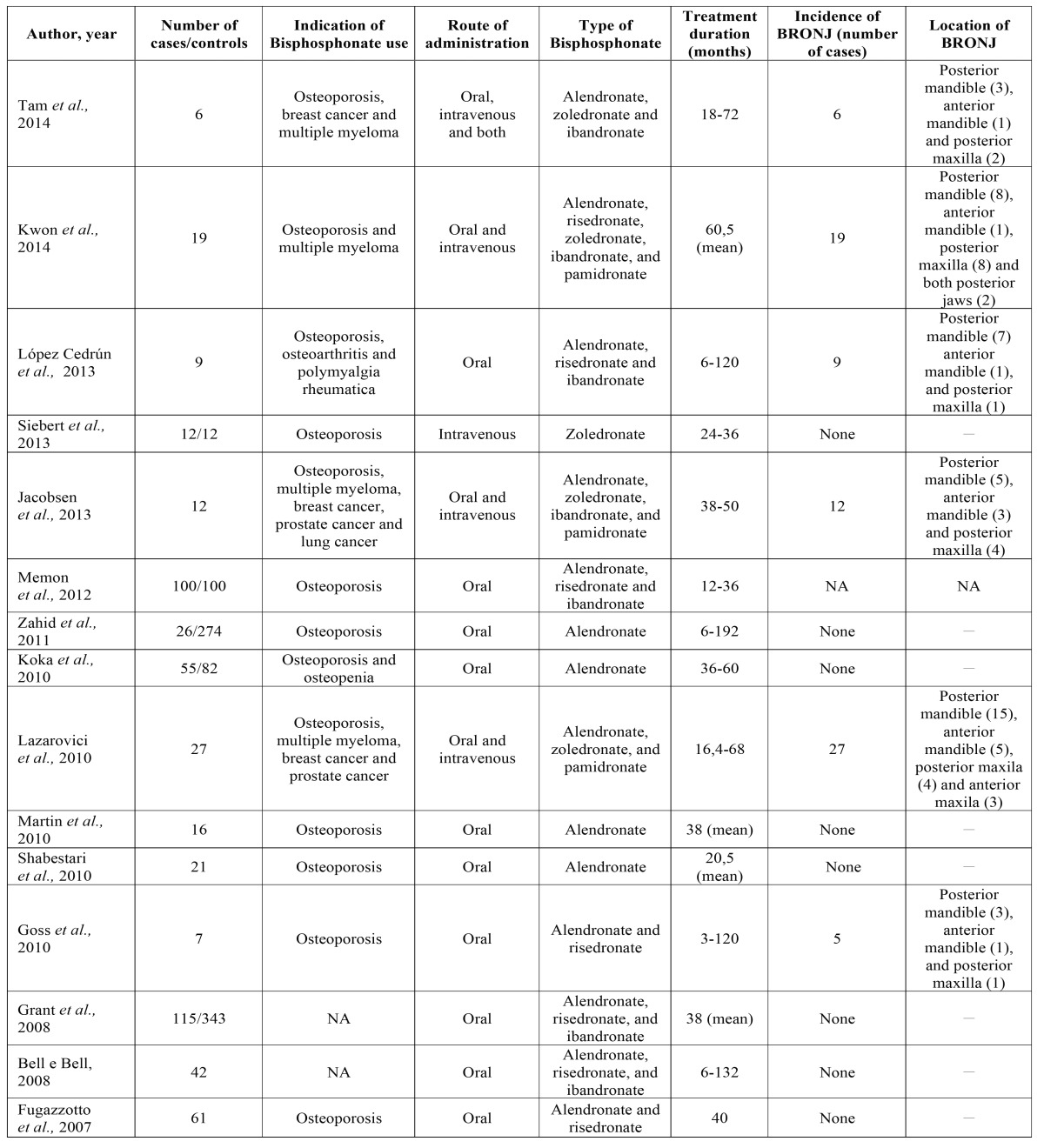
Summary of the studies presenting data about bisphosphonate related osteonecrosis of the jaws (BRONJ).

- Studies description

Eight were retrospective studies (8-15) one was prospective study (16) and six were case series (17,3,18-21). The articles were classified according to the levels of evidence (based on the University of Oxford’s Center for Evidence Based Medicine criteria) (22) ( Table 3).

**Table 3:**
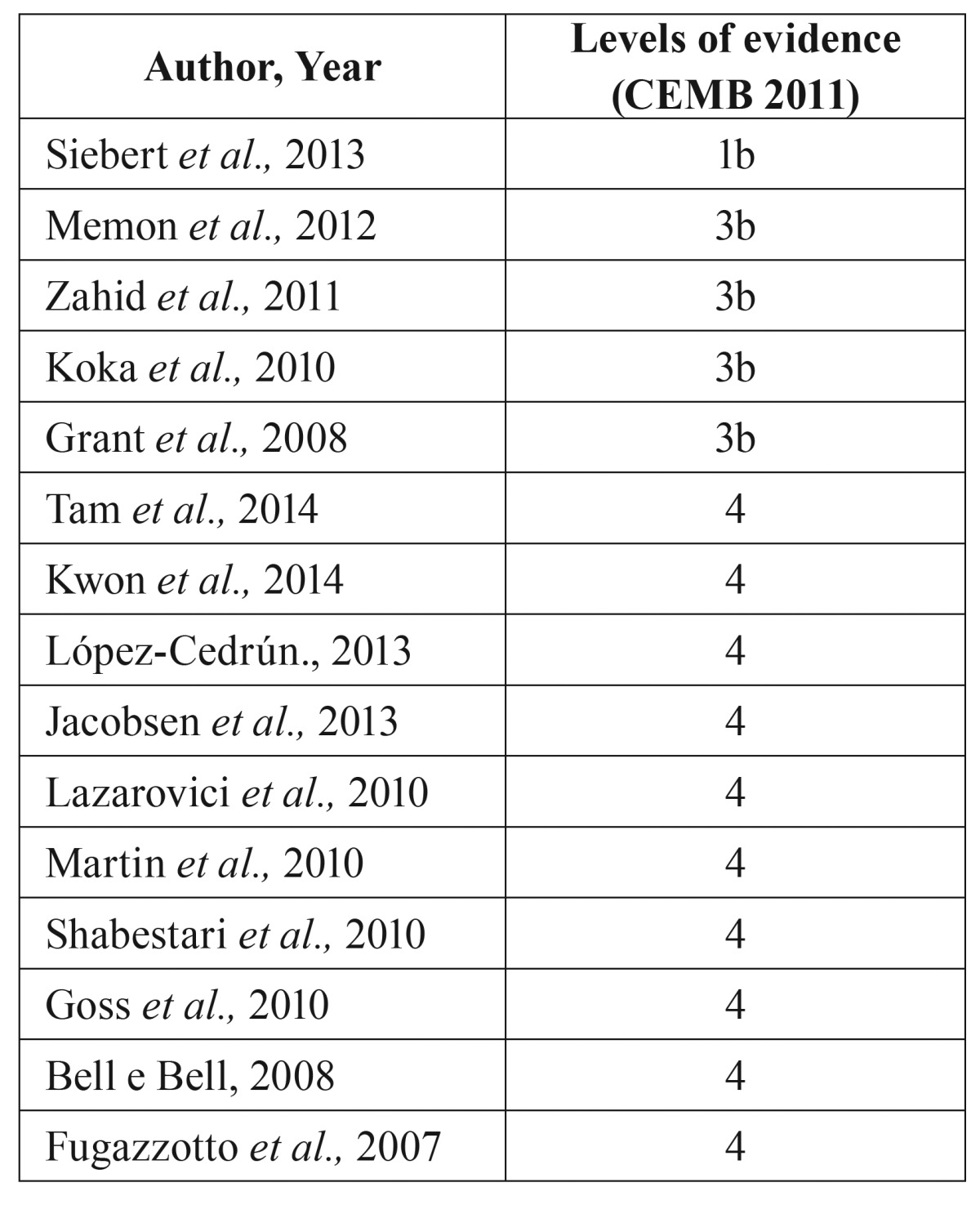
Levels of clinical evidence (CEBM 2011).

Overall, this systematic review analyzed 1339 patients (528 patients with a history of BP use and 811 patients without history of BP) with 3748 implants placed (1330 in BP users and 2418 in control patients) and 152 loss of implants (113 in BP users and 39 in control patients). Patient´s age ranged from 17 to 91 years and most of them were female gender. There were 78 cases of osteonecrosis and the lesions occurred in mandible (53 cases), maxilla (23 cases) and 2 in both jaws. The majority of the lesions were located predominantly in the posterior areas (63 cases). Follow-up period ranged from 1 to 132 months.

Ten of the studies selected presented bisphosphonate therapy administered orally (alendronate, risedronate, ibandronate) (17,3,18,8-10,12-14,20), 4 both (alendronate, risedronate, pamidronate, zolendronate and ibandronate) (11,19,15,21) and 1 intravenously (zoledronate) (16). Among studies which have reported oral route of administration of bisphosphonate, only two (18,20) related cases of osteonecrosis. On the other hand, one hundred percent of the studies (11,19,15,21) which related combined use of oral and intravenous BP, have shown cases of osteonecrosis. Duration of BP therapy ranged from 3 to 192 months. Osteoporosis and malignant diseases were the most commonly indication for BP use.

## Discussion

Given the widespread use of bisphosphonates for several conditions and the large use of dental implants for treatment of partial or complete edentulism, as well as the increasing of cases of bisphosphonate related osteonecrosis of the jaw, it is of matter importance to evaluate the relation between these topics to find out the risks for the osseointegration process and BRONJ appearance.

To Holzinger *et al.* (23), the development of osteonecrosis in conjunction with dental implants might be a side effect of treatment with oral or intravenous BPs. The incidence of BRONJ is accelerated after the conclusion of, or during, BP therapy. From their data, BPs could have a potentiating effect on peri-implantitis and implant loss.

Javed and Almas (24) showed that the incidence of implant failure was minimal in patients using oral and intravenous bisphosphonates, and concluded that dental implants in patients undergoing BPs therapy can osseointegrate and remain functionally stable. On the other hand, Mínguez-Serra *et al.* (25) suggested the avoidance of dental implant procedures in patients that have been receiving intravenous BPs. This is in accordance with the results of the present review on where one hundred percent of the studies (11,19,15,21) which related combined use of oral and intravenous BP, have shown cases of osteonecrosis. In the case of administration via oral route, caution is required, avoiding these procedures, or indicating them only when absolutely necessary.

Bell and Bell (13) had a success rate of 95% in 100 dental implants installed in 42 patients taking oral bisphosphonates and they did not present signs of osteonecrosis of the jaws. Therefore, they concluded that there is no relationship between oral medications containing BPs and implant failure. Others authors (3,8,9,10,14) suggested that bisphosphonates exposure and implant placement do not affect implant success and do not result in osteonecrosis. However, their duration follow-up was short. These results are in accordance with other publications (24,26).

The study of Yip *et al.* (27) indicates that women with implant failure had increased odds of reporting a history of oral bisphosphonate use compared with those without implant failure. These findings suggest that dental practitioners should be aware of the increased risk of implant failure associated with oral bisphosphonate use in certain patient populations. Their conclusion is in agreement with the recommendation for discontinuation of oral bisphosphonate therapy in long-term oral bisphosphonate users for 4-6 months prior to implant insertion, and several months after, to allow for the recovery of bone remodeling (28).

Lazarovici *et al.* (19) followed 27 patients who developed BRONJ associated with dental implants and concluded that this condition is a side effect of BPs treatment presented like a late complication. They suggested that patients undergoing bisphosphonate therapy who receive dental implants should be followed for long periods, and those ones who developed BRONJ associated with dental implants should undergo a long-term treatment with doxycycline 100 to 200 mg/d, and their dental implants should be removed only if the antibiotic therapy fails to alleviate the signs and symptoms of BRONJ. The duration of bisphosphonate treatment in the studies with cases of BRONJ ranged from 3 months up to 120 months (reaching more than four years in the most), therefore all of them have shown long duration of treatment. This information might be related to the fact that, as Lazarovici *et al.* (19) have shown, the osteonecrosis is a late complication, thus the follow up period must be extended in order to find late signs and symptoms.

The literature reviewed say that patients who take oral bisphosphonates, can be submitted to dental implant surgery, on the condition that the risks are thoroughly assessed. The evaluation of the risks associated to the patients comprises: type of agent, dose, and duration of BP treatment (determinant); female gender, age greater than 65 years, comorbidities such as diabetes or obesity, tobacco abuse, concomitant treatment such as corticotherapy, chemotherapy, immunosuppressive therapy, mandibular localization, posterior area, bone diseases such as exostosis, or tori, harboring a badly fitted prosthesis (potentially aggravating), and periodontal disease, bad oral and dental hygiene (aggravating) (29).

Diabetes, chemotherapy, steroids use, hypertension and smokers habits were the most common risk factors found among the patients enrolled on the studies. Implant supported dentures are great resources to rehabilitation of edentulous areas in comparison with the traditional prosthetic appliances, however the bone condition (quantity and quality) and its healing capacity are factors that cannot be left without the appropriate attention because they can influence the success rate of the dental implants procedures.

The most of the studies (18,11,19,15,21) with cases of osteonecrosis enrolled patients with underlying disease such as malignant diseases, osteoarthritis and polymyalgia rheumatic as indication, whereas the majority (7,3,10,12,16) of the studies with no cases of osteonecrosis, presented only osteoporosis as indication for BP therapy. This information suggests that general health status of the patients might also have contributed with the development of BRONJ.

Some authors suggest the use of the Telopeptide C terminal CTX Test as a method to define the risk of development of osteonecrosis of the jaws in patients undergoing bisphosphonate therapy by measuring a specific crosslink peptide of type I collagen in bone (30,28). However, it is important to note that recent guidelines do not consider such method neither validated nor recommended and it has not been advisable its use (29,5).

According to the AAOMS (5), individuals who have taken oral BP for less than four years and have no risk factors, do not need any alteration in the planned surgery. If a dental implant surgery is proposed, informed consent should be provided reporting possible long-term implant failure and the low risk of developing osteonecrosis of the jaws. Such patients should be assessed on a regular dental follow-up. For those patients who have taken oral BP for less than four years and have also taken corticosteroids or antiangiogenic medications concomitantly, or for patients who have taken oral BP for more than four years with or without any concomitant medical therapy, the discontinuation of this drugs (drug holiday) should be considered for at least two months prior to surgery, if systemic conditions permit and bisphosphonate should not be restarted until osseous healing has occurred.

Dentoalveolar surgery is considered great risk factor for the Medication Related Osteonecrosis of the jaw (MRONJ). It is reported that among patients with MRONJ 52 to 61% of patients report tooth extraction as the precipitating event (5). Above all, it is of matter importance to be aware of the great destructive potential of osteonecrosis of the jaws. These lesions can cause large deformity in the face of the patients. The BRONJ can result in significant functional and aesthetic defects since the treatment usually involves debridement and resection of the affected area.

This study analyzed 528 patients with history of BP use, with 1330 implants placed in these patients. There were 113 loss of implants (8.49%) in BP users and 78 cases of osteonecrosis (14.77%). These results show high percentages of loss of implants and notably an elevate incidence of osteonecrosis. Considering these data, it is reasonable to be cautious during the planning of implant surgery for patients undergoing bisphosphonate therapy. And going beyond, maybe the health professionals should start to indicate dental procedures such as dental prophylaxis, restorations, gingival curettage, root scaling, endodontic treatments and extractions before the patients initiate the bisphosphonate therapy with the goal of avoid invasive dental procedures during the BP treatment, likewise it is done with patients who are going to be submitted to radiotherapy.

- Study limitation

The main limitation of our study is the lack of randomized clinical trial related to the theme, which limits the level of evidence of the obtained information. Moreover, a meta-analysis was not possible to because of the heterogeneity of the studies and their presented data.

Conclusions

Considering the limitations of this study, it is wise to be careful when comes the time of planning dental implant surgery in patients undergoing bisphosphonate therapy. The risk of developing BRONJ as well as occurring failure or loss of implant exists and it is greater in patients under intravenous bisphosphonate therapy. A complete medical history of the patient must be analyzed and in the case of the therapy with bisphosphonate be confirmed, the duration of treatment, as well as the route of administration should be taking into consideration. Then, if possible, suspend the treatment based on the AAOMS recommendation. Ultimately, further randomized clinical trials with longer follow-up period are needed because it remains unclear in what intensity the exposure to these medications is harmful to implant treatment.
